# Systemic Induction of NO-, Redox-, and cGMP Signaling in the Pumpkin Extrafascicular Phloem upon Local Leaf Wounding

**DOI:** 10.3389/fpls.2016.00154

**Published:** 2016-02-12

**Authors:** Frank Gaupels, Alexandra C. U. Furch, Matthias R. Zimmermann, Faxing Chen, Volkhard Kaever, Anja Buhtz, Julia Kehr, Hakan Sarioglu, Karl-Heinz Kogel, Jörg Durner

**Affiliations:** ^1^Institute of Biochemical Plant Pathology, Helmholtz Zentrum München, German Research Center for Environmental HealthNeuherberg, Germany; ^2^Institute of General Botany and Plant Physiology, Friedrich-Schiller-UniversityJena, Germany; ^3^College of Horticulture, Fujian Agriculture and Forestry UniversityFuzhou, China; ^4^Research Core Unit Metabolomics, Hannover Medical SchoolHannover, Germany; ^5^Department Lothar Willmitzer, Max Planck Institute of Molecular Plant PhysiologyPotsdam, Germany; ^6^Biocenter Klein Flottbek, University HamburgHamburg, Germany; ^7^Department of Protein Science, Helmholtz Zentrum München, German Research Center for Environmental HealthNeuherberg, Germany; ^8^Research Center for BioSystems, Land Use and Nutrition, Institute of Phytopathology, Justus Liebig University GiessenGiessen, Germany

**Keywords:** phloem, systemic, wound response, signaling, NO, redox, antioxidant system, cGMP

## Abstract

Cucurbits developed the unique extrafascicular phloem (EFP) as a defensive structure against herbivorous animals. Mechanical leaf injury was previously shown to induce a systemic wound response in the EFP of pumpkin (*Cucurbita maxima*). Here, we demonstrate that the phloem antioxidant system and protein modifications by NO are strongly regulated during this process. Activities of the central antioxidant enzymes dehydroascorbate reductase, glutathione reductase and ascorbate reductase were rapidly down-regulated at 30 min with a second minimum at 24 h after wounding. As a consequence levels of total ascorbate and glutathione also decreased with similar bi-phasic kinetics. These results hint toward a wound-induced shift in the redox status of the EFP. Nitric oxide (NO) is another important player in stress-induced redox signaling in plants. Therefore, we analyzed NO-dependent protein modifications in the EFP. Six to forty eight hours after leaf damage total S-nitrosothiol content and protein S-nitrosylation were clearly reduced, which was contrasted by a pronounced increase in protein tyrosine nitration. Collectively, these findings suggest that NO-dependent S-nitrosylation turned into peroxynitrite-mediated protein nitration upon a stress-induced redox shift probably involving the accumulation of reactive oxygen species within the EFP. Using the biotin switch assay and anti-nitrotyrosine antibodies we identified 9 candidate S-nitrosylated and 6 candidate tyrosine-nitrated phloem proteins. The wound-responsive Phloem Protein 16-1 (PP16-1) and Cyclophilin 18 (CYP18) as well as the 26.5 kD isoform of Phloem Protein 2 (PP2) were amenable to both NO modifications and could represent important redox-sensors within the cucurbit EFP. We also found that leaf injury triggered the systemic accumulation of cyclic guanosine monophosphate (cGMP) in the EFP and discuss the possible function of this second messenger in systemic NO and redox signaling within the EFP.

## Introduction

Leaf damage by insects, herbivores or abiotic sources triggers wound responses in plants (Savatin et al., 2014). Affected tissues rapidly cumulate defensive proteins, protease inhibitors and secondary metabolites for fighting off herbivorous attackers or microbial invaders. Subsequently, the wound site is sealed by induction of localized cell death and callose formation. These responses are concerted amongst others by the general stress messengers hydrogen peroxide (H_2_O_2_), nitric oxide (NO) and the more damage-specific phytohormone jasmonic acid (JA) (Orozco-Cárdenas et al., 2001; Wünsche et al., 2011; Zebelo and Maffei, 2015). Interactions of NO and H_2_O_2_ with calcium and cyclic guanosine monophosphate (cGMP) have been frequently reported although little is known about a role of cGMP in wound signaling (Lin et al., 2011, 2012).

Plants do not only react locally to a threat but signals are often translocated to distant plant parts which are thus prepared for the upcoming stress encounter. Several current reports suggested functions of calcium, calcium-dependent electric signals, H_2_O_2_-autopropagation waves, NO and JA in the plant systemic wound response (SWR) (Mousavi et al., 2013; Gilroy et al., 2014; Zebelo and Maffei, 2015). However, the exact sequence and mode of interaction between these signals remains to be deciphered, and transport routes are also not well defined for all signals. The phloem distributes assimilates from autotrophic to heterotrophic plant parts and was shown to be involved in signal transport during SWR. For instance, JA and NO derivatives were detected within the vasculature after leaf wounding (Glauser et al., 2009; Chaki et al., 2011; Espunya et al., 2012; Furch et al., 2014) while electrical signals spread in a pattern, which was at least to some extent dependent on phloem connectivity but independent of assimilate flow (Salvador-Recatalà et al., 2014). Micromolar concentrations of H_2_O_2_ induced within seconds calcium-dependent NO production in the phloem of *Vicia faba* (Gaupels et al., 2008). Taken together these findings argue for complex defense signaling within the phloem.

Due to its high nutrient content the phloem is an attractive target for insect and pathogen attack and therefore disposes of effective defense mechanisms. Cucurbits have developed the unique extrafascicular phloem (EFP), which forms a specialized defense structure against herbivores similar to laticifers in other plant species (Konno, 2011; Gaupels and Ghirardo, 2013). Exudates from the EFP contain toxic cucurbitacin steroids, alkaloids and terpenoids as a preformed barrier against invaders (Konno, 2011). Moreover, metabolomic and proteomic approaches revealed that leaf damage triggered SWR in the EFP of *Cucurbita maxima* (pumpkin) amongst others by JA and redox signaling (Gaupels et al., 2012).

In the present study, we aimed at further exploring signal transduction induced in the EFP during systemic wound responses. We were particularly interested in alterations of the antioxidant system as a hint toward induced redox changes and in signaling by NO-mediated protein modifications and cGMP. The observed damage responses might be transmitted over long distances via the phloem or could be part of EFP-internal defense mechanisms triggered by systemic messengers.

## Materials and methods

### Plant treatment and sampling

Leaf edges of 4–5 week-old pumpkin plants (*Cucurbita maxima* cv. Gele Centenaar) grown under green-house conditions were crushed between the lids of two 50 ml polypropylene reaction tubes. Control plants were left untreated. Phloem sap was collected as described earlier (Gaupels et al., 2012). Petioles and stems were cut using a razor blade and the basal side of the cut was immediately blotted with tissue paper. The exuding phloem sap was subsequently collected by a micropipette and mixed with an equal volume of phloem buffer (50 mMTris/HCl, pH 7.8, 0.1% β-mercaptoethanol; McEuen and Hill, 1982). Pumpkin leaf extracts were prepared by grinding 0.5 g leaf material in liquid N_2_, addition of 3 ml homogenization buffer (50 mM TrisCl, pH 7.8, 1 mM EDTA, 7.5% [w/v] soluble polyvinylpyrrolidone, 2 mM ascorbate) and subsequent centrifugation. The supernatant was used for APX measurements.

### Measurements of antioxidant enzymes, glutathione and ascorbate

All enzyme measurements were done with an Ultrospec 3100 Pro photometer (GE Healthcare Life Sciences) following previously published protocols (Harrach et al., 2008). APX activity was measured in 36 μl phloem sample (phloem exudate plus phloem buffer) or 50 μl leaf extract while 10 and 32 μl aliquots of phloem samples were used for determination of DHAR and GR activities, respectively. For the glutathione and ascorbate measurements 10 μl phloem exudate was added to 90 μl of 5% meta-phosphoric acid. Samples were incubated for 10 min at RT and centrifuged for 30 min at 14000 rpm. The supernatant was stored at −20°C until further analysis. Immediately before the measurements samples were neutralized by adding 25 μl 1 M triethanolamine. Glutathione was measured in 5 μl neutralized extract using the Amplite™ Fluorimetric Glutathione GSH/GSSG Ratio Assay Kit (AAT Bioquest) following the manufacturer's instructions.

For ascorbate measurements, a colorimetric protocol was used (Harrach et al., 2008). Five microliter neutralized phloem extract was mixed with 150 μl 150 mM NaPO_4_ (pH 7.4) and 150 μl H_2_O_dest_ to determine reduced ascorbate. For the measurement of total ascorbate neutralized extract was mixed with 150 μl 150 mM NaPO_4_ and 75 μl 10 mM dithiothreitol. After 10 min incubation at RT 75 μl 0.5% N-ethylmaleimide was added to the sample. The reaction protocol is the same for both reduced and total ascorbate. The sample was combined with 300 μl 10% (w/v) trichloroacetic acid, 300 μl 44% (v/v) phosphoric acid, 300 μl 4% (w/v) bipyridyl (in 70% EtOH), and 150 μl 2% (w/v) FeCl_3_. After 1 h incubation at 37°C the absorption of the sample was measured at 525 nm.

### Thiobarbituric acid reactive substances determination

Proteins were removed from 100 μl phloem exudate by adding 200 μl ice-cold trichloroacetate, incubation for 15 min on ice and subsequent centrifugation. Two hundred fifty microliters of the supernatant was used for determining the content of thiobarbituric acid reactive substances (TBARS) according to Hodges et al. (1999).

### Determination of the total S-nitrosothiol content

The total S-nitrosothiol content of phloem sap was analyzed by a Nitric Oxide Analyzer (Siever's NOA 280i, GE Power and Water, Analytix). Seventy five microliters phloem exudate was treated for 10 min at RT with 19 μl 5% sulfanilamide (w/v, in 1 M HCl) in order to scavenge nitrite. The sample was then injected into the NOA reaction vessel which contained a reducing triiodide solution (Piknova and Schechter, 2011). Released NO reacted with ozone generated by the NOA and the resulting chemiluminescence was recorded by an internal detector. Peak area integration and calculation of SNO concentrations based on nitrite standards were performed using the Siever's NO Analysis Software.

### Investigation of protein S-nitrosylation by the biotin switch assay

S-nitrosylation of protein Cys residues was investigated by the biotin switch method (Jaffrey and Snyder, 2001). This approach is based on blocking free Cys residues by methanethiosulfonate (MMTS) or iodoacetamide, breaking of NO-Cys bonds by ascorbate and subsequent attachment of biotin to formerly S-nitrosylated Cys. Biotinylated proteins can then be detected in WB analysis or isolated using streptavidin-coated magnetic beads. For WB analyses phloem proteins were irreversibly alkylated by iodoacetamide which prevented sample gelation through redox-dependent polymerization of PP1 and PP2. For this, 1 volume of EFP exudate was mixed with 2 volumes of 200 mM iodoacetamide in HEN (25 mM HEPES/NaOH, 1 mM EDTA, 0.1 mM neocuproin, pH 7.7)/1% sodium dodecyl sulfate (SDS) and then incubated for 30 min at room temperature. Proteins were precipitated with ice-cold acetone, re-dissolved in 1 volume (of the original phloem sample) 1 mM ascorbate/1 mM Biotin-HPDP (Pierce) and incubated for 1 h at RT for de-nitrosylation and subsequent biotinylation. After another acetone precipitation proteins were dissolved in non-reducing loading buffer, separated by SDS-PAGE, transferred to a nitrocellulose membrane by semi-dry blot and finally immuno-detected by an monoclonal anti-biotin antibody coupled to alkaline phosphatase (Sigma) using a commercial substrate.

PP1 and PP2 are highly abundant in phloem sap masking other phloem proteins in down-stream analyses. Therefore, it was necessary to remove these proteins before isolation and identification of S-nitrosylated phloem proteins by MS. Polymerization of the redox-sensitive PP1 and PP2 was induced by adding 400 μl alkaline HEN buffer to 100 μl phloem exudate (Alosi et al., 1988). The PP1 and PP2 containing gel-like matrix was removed by a pipet tip and the liquid phase was further analyzed. One hundred microliters sample was incubated for 45 min at RT with 400 μl 10 mM GSH for removing SNO groups or GSNO for *in vitro* S-nitrosylation of amenable Cys residues. Free Cys residues were blocked by adding 50 μl 25% SDS and 1.5 μl 10.6 M MMTS (in dimethylformamide) followed by 1 h incubation at 50°C with regular vortexing. Proteins were precipitated with 2 volumes ice-cold acetone. S-nitrosylated proteins were biotinylated by dissolving the precipitated proteins in 20 μl 1 mM ascorbate/1 mM Biotin-HPDP (Pierce) and incubation for 1 h at room temperature. After another acetone precipitation the resulting pellet was dissolved in 200 μl HEN/1% SDS and 400 μl neutralization buffer (20 mM HEPES/NaOH; 1 mM EDTA; 100 mM NaCl; 0.5% Triton X-100; pH 7.7).

We used streptavidin-coated magnetic beads (Dynabeads, Invitrogen) for isolation of biotinylated proteins according to the manufacturer's instructions. All washing steps were done with neutralization buffer. Biotinylated proteins were eluted by incubating the beads in 4 × Laemmli buffer for 5 min at 90°C. SDS-PAGE, colloidal coomassie staining, and the determination of partial amino acid sequences were performed as described in Walz et al. (2002). In short, gels were stained with colloidal Coomassie stain (Novex) over night and destained for 2 h in water. Protein bands were excised, destained, dehydrated, and digested overnight with modified trypsin (Roche Diagnostics). Peptides were extracted and vacuum-dried. Desalted digests were analyzed by an electrospray ionization quadrupole time-of-flight tandem mass spectrometer (Q-TOF, Micromass/Waters). After data processing and analysis of the resultant peptide fragmentation data, database similarity searches with the derived sequence stretches were performed with the short sequence blast algorithm (http://www.ncbi.nlm.nih.gov/blast/) against the non-redundant protein database, limited to green plants.

### Investigation of tyrosine nitration

For WB analyses we loaded 0.5 μl sample in 50 μl phloem buffer on a nitrocellulose membrane using a 96-well vacuum dot-blot device (Bio-Dot, BioRad). Equal loading was checked by Ponceau Red staining of the blot membrane before blocking with 1% (w/v) milk powder. The primary antibody against nitrotyrosine (monoclonal anti-nitrotyrosine antibody, mouse, clone 1A6, Upstate) was diluted 1:1000 and the second antibody (anti-mouse-horse raddish peroxidase conjugate, goat, Invitrogen) was diluted 1:20000. After addition of the chemiluminescent peroxidase substrate, X-ray films were exposed to the blot membranes for 10–20 s and developed.

Tyrosine nitrated phloem proteins were immunoprecipitated using an anti-nitrotyrosine monoclonal antibody coupled to agarose (clone 1A6, Cayman). Fifty microliters phloem sample (exudate plus buffer) was diluted with 450 μl PBS containing 0.05% Tween-20. After adding 25 μl of antibody suspension the sample was incubated for 2 h at RT under agitation, centrifugated and 3 times washed with PBST. Finally, bound proteins were eluted by 5 min incubation at 95°C in 25 μl denaturating Laemmli buffer. SDS-PAGE and silver staining procedures were like previously described (Gaupels et al., 2012). For identification of tyrosine nitrated EFP proteins silver stained bands were manually cut, destained and washed with buffer containing 50 mM NH_4_HCO_3_ in 30% acetonitrile (ACN) and equilibrated in 10 mM NH_4_HCO_3_ prior to proteolytic digestion. Gel pieces were shrunk with 100% v/v ACN and rehydrated in 10 mM NH_4_HCO_3_. This treatment was repeated, followed by the addition of 0.1–0.2 μg of modified trypsin (SIGMA) per piece. Digestion was carried out overnight at 37°C. The supernatant was collected and combined with the eluates of subsequent elution steps with 80% v/v ACN, 1% v/v TFA. The combined eluates were dried in a SpeedVac centrifuge. The dry samples were dissolved in 20 μl 50% v/v ACN, 0.1% v/v TFA for the subsequent MALDI preparation. Therefore, 0.5 μl of a 1:1 mixture of sample and a matrix solution consisting of 5 mg/mL CHCA (Bruker, Bremen, Germany) were spotted on a MALDI target.

Mass spectra were acquired using a Proteomics Analyzer 4700 (MALDI-TOF/TOF) mass spectrometer (Applied Biosystems, Framingham, USA). Measurements were performed with a 355 nm Nb:YAG laser in positive reflector mode with a 20 kV acceleration voltage. For each MS and MS/MS spectrum 3000 shots were accumulated. For each spot on a MALDI plate the eight most intense peptides were selected for additional MS/MS analysis. The acquired MS/MS spectra were searched with protein pilot software 3.0 against the Swiss-Prot database (updated August 2010; 519348 sequences, 183273162 residues) using an in-house version of Mascot 2.3.02. The resulting peptide fragmentation data were blast searched (http://www.ncbi.nlm.nih.gov/blast/) against the non-redundant protein database, limited to green plants.

### cGMP measurements

cGMP was extracted from 150 μl phloem sap by adding 300 μl EtOH followed by an incubation for 10 min at room temperature. After centrifugation the supernatant was vacuum-dried and stored at −20°C until further analysis. cGMP measurements by the cGMP Enzyme Immunoassay Kit (Sigma-Aldrich) were done according to the manufacturer's instructions. Reversed Phase chromatographic separation of 3′,5′-GMP was performed using a Shimadzu HPLC-system (Shimadzu). A Zorbax eclipse XCB-C18 1.8 μm column (50 × 4.6 mm; Agilent) connected to a C18-Security guard (Phenomenex) and a 2 μm column saver, kept at 25°C, was applied. The mobile phases were 3/97 methanol/water [v/v] (A) and 97/3 methanol/water [v/v] (B), each containing 50 mM ammonium acetate and 0.1% acetic acid. The following gradient was applied: 0–5 min, 0–50% B, and 5–8 min 0% B. The flow rate was 500 μL/min. Detection and quantification of cyclic nucleotides was carried out by a tandem mass spectrometer, 5500Q TRAP (AB Sciex), equipped with an electrospray ionization source, operating in positive ionization mode.

## Results and discussion

### The antioxidant system in the EFP is systemically down-regulated after leaf wounding

**Figure 1:**
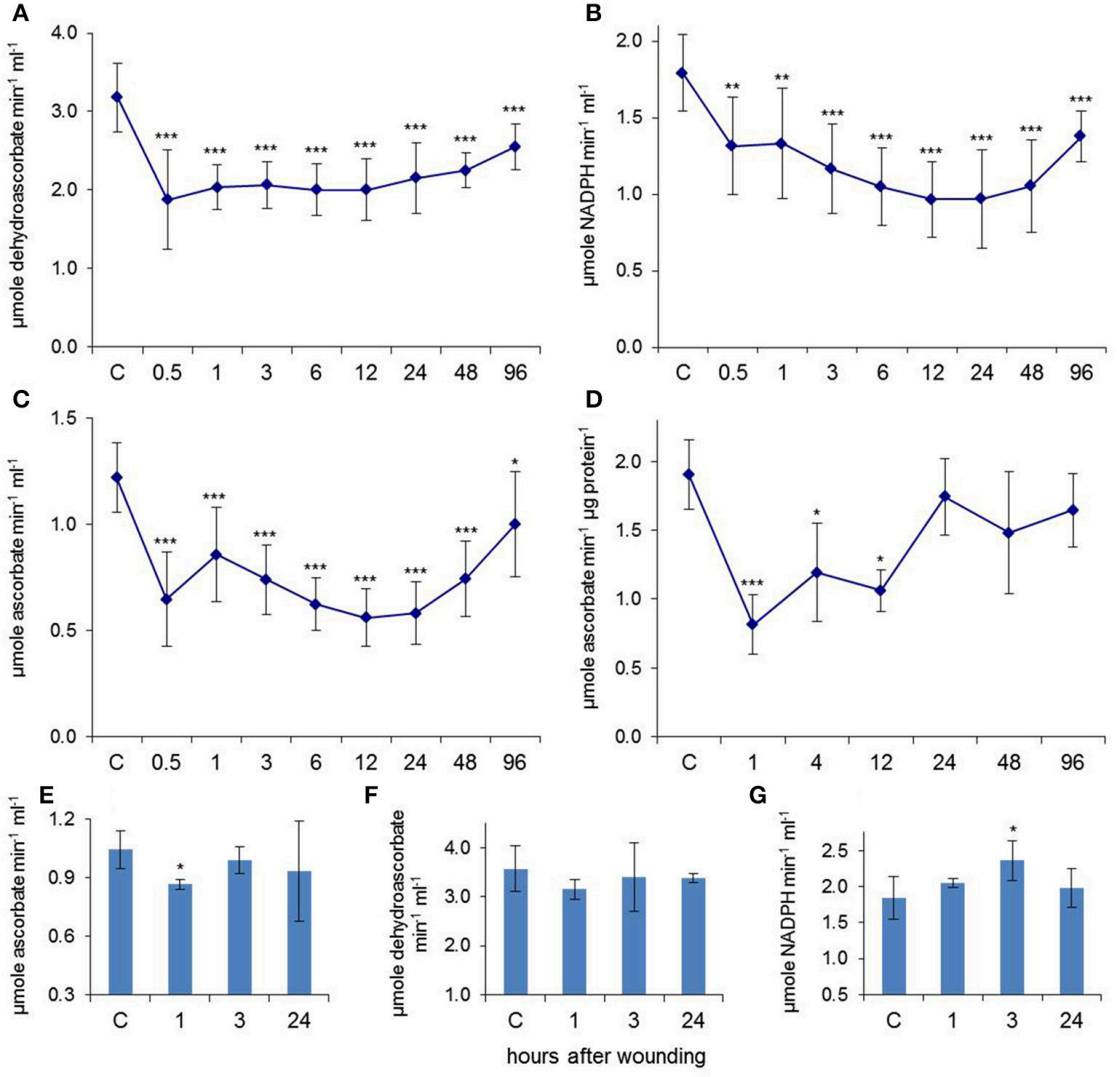
**Leaf wounding induced systemic regulation of antioxidant enzymes in the EFP**. Enzyme activities of **(A)** DHAR, **(B)** GR, and **(C)** APX decreased in phloem exudates after wounding of all leaves. Data points represent means (±SD, *n* = 9–12). **(D)** APX activity was also reduced in extracts from injured leaf tissues (*n* = 5). **(E–G)** Systemic regulation of APX, DHAR, and GR after wounding solely the two lowest leaves. Columns represent means (±SD, *n* = 5). Asterisks indicate significant differences from control (C) samples (Student's *t*-test, ^*^p < 0.05, ^**^p < 0.01, ^***^p < 0.005).

The EFP has a complete antioxidant system including various antioxidant enzymes and high levels of glutathione and ascorbate (Alosi et al., 1988; Walz et al., 2002; Lin et al., 2009). We focussed our work on ascorbate peroxidase (APX), dehydroascorbate reductase (DHAR), and glutathione reductase (GR) because they represent core elements of the Foyer-Halliwell-Asada cycle and displayed robust activities in phloem sap. All three investigated enzymes were significantly down-regulated already at 30 min after leaf squeezing (Figures 1A–C). Inhibition was most pronounced after 30 min for DHAR but much later at the 24 h time point for GR. APX underwent a bi-phasic depression of activity with minima at 0.5 and 24 h after wounding. Only an early drop in APX activity was observed in extracts from wounded leaf tissues (Figure 1D) suggesting that the bi-phasic inhibition of this enzyme was specific for the EFP.

The APX activity in phloem samples decreased by up to 54% when all leaves were wounded, whereas APX was down-regulated by 17% when just the two lowest leaves were wounded (Figure 1E). DHAR was not significantly affected (Figure 1F) whereas the GR activity was even increased by 28% at 3 h after the local stress treatment (Figure 1G). The results confirm that EFP responses to leaf damage are (1.) systemically transduced over long distances as well as (2.) dose- and/or distance-dependent in nature. It was previously observed that APX and catalase activities were transiently down-regulated in total leaf extracts of *Pelargonium peltatum* already at 5 and 8 h after leaf wounding, which caused an accumulation of H_2_O_2_ (Arasimowicz et al., 2009). Decreasing the antioxidant capacity of damaged and adjacent tissues is probably a mechanism for modulating defense signaling and wound healing by H_2_O_2_. The kinetics of APX inhibition are remarkably similar between pelargonium leaves, pumpkin leaves, and pumpkin EFP suggesting that down-regulation of antioxidant enzymes is part of a general plant response to acute stress.

**Figure 2:**
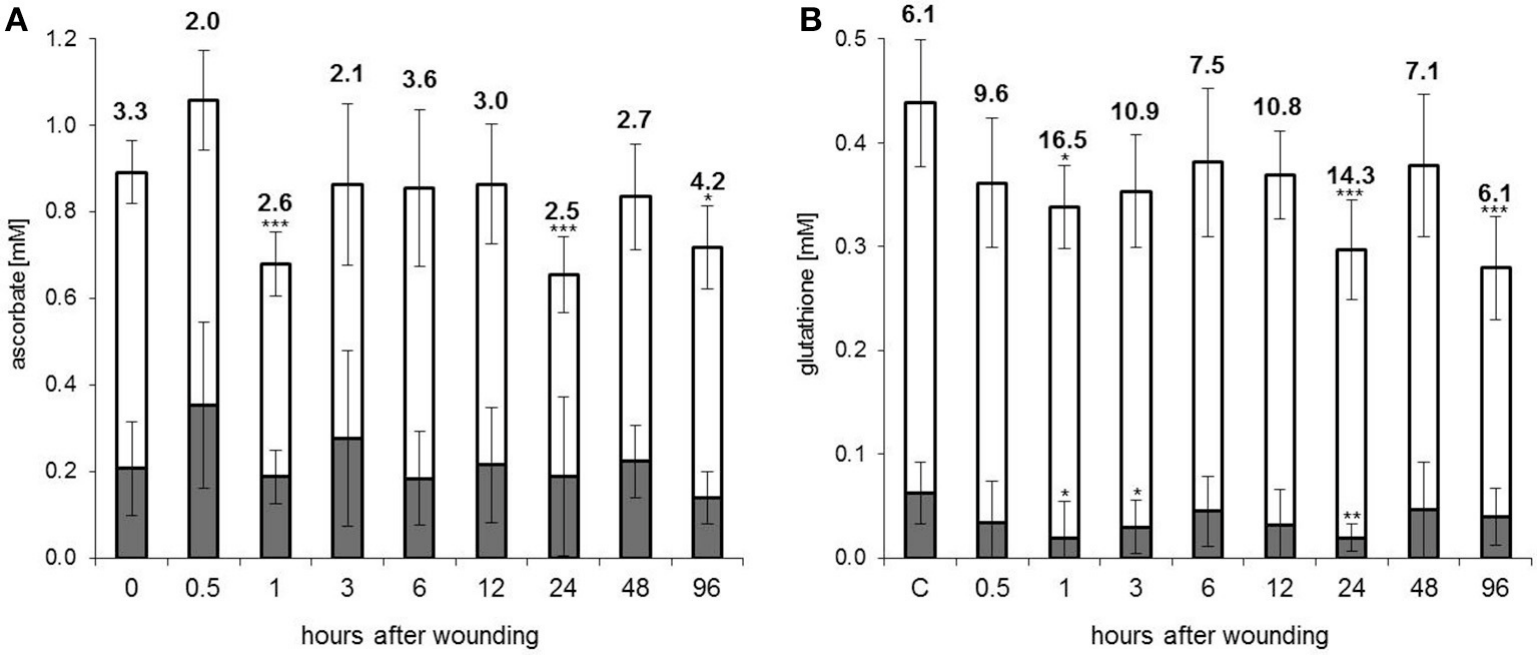
**Antioxidant levels in the EFP are decreased after leaf damage. (A)** Ascorbate and **(B)** glutathione concentrations in phloem latex. White sections of the columns represent the reduced fractions of ascorbate (AsA) and glutathione (GSH). Oxidized ascorbate (DHA) and glutathione (GSSG) are shown in gray. Numbers are the AsA/DHA and GSH/GSSG ratios. Columns represent means (±SD, *n* = 7–9). Asterisks indicate significant differences from control (C) samples (Student's *t*-test, ^*^p < 0.05, ^**^p < 0.01, ^***^p < 0.005).

A long-lasting reduction in antioxidant enzyme activities in the EFP would likely cause accumulation of reactive oxygen species (ROS) from different sources including energy metabolism and ROS generating enzymes. Under these conditions reduced glutathione (GSH) and ascorbate (AsA) would be oxidized to give glutathione disulfide (GSSG) and dehydroascorbate (DHA). Although both GSH as well as AsA levels were diminished after wounding there was no obvious accumulation of GSSG and DHA (Figures 2A,B). GSSG was even decreased whereas DHA levels were not significantly changed. The AsA/DHA ratio, which is a measure for the redox potential of the ascorbate pool, was lower at 0.5–3 h and 24 h after wounding whereas the GSH/GSSG ratio was even increased at these time points. Thus, rather than a consistent shift in GSH/GSSG and AsA/DHA ratios we observed a decline in total glutathione and ascorbate levels. Twenty four and 27% of free ascorbate but 23 and 32% of free glutathione disappeared from the EFP at 1 and 24 h after leaf damage. The wound-induced decrease in total ascorbate content is a common phenomenon reported for various plants (Suza et al., 2010). The bi-phasic drop in antioxidants matched well with the down-regulated activities of DHAR, GR and APX at these time points. The fate of antioxidants under severe stress conditions is not fully resolved but it is known that inhibition of GR and DHAR prevents recycling of AsA and GSH from DHA and GSSG. DHA is rather unstable and rapidly decays amongst others to oxalate and tartrate while GSSG binds to oxidized proteins by S-glutathionylation (Noctor and Foyer, 1998).

### ROS in the EFP

Down-regulation of the antioxidant system often correlates with ROS accumulation and subsequent oxidative modifications of proteins and lipids. However, using four different assays based on Amplex Red, H_2_DCF (Harrach et al., 2008), Xylenol Orange/sorbitol (Gay and Gebicki, 2000) or dimethylaminobenzoic acid (DMAB) in conjunction with methylbenzothiazoline hydrazone (MBTH) (Okuda et al., 1991; Veljovic-Jovanovic et al., 2002) we did not detect ROS in the EFP after leaf squeezing. There was no general rise in protein oxidation while carbonylation of PP2 was even transiently decreased at 6–24 h after wounding (Gaupels et al., 2012). Moreover, formation of malondialdehyde as an indicator for lipid oxidation and oxidative stress did not show strong alterations (Supplemental Figure 1). From these results it can be concluded that ROS do not accumulate to detectable levels in the EFP of stressed pumpkin. Most likely, ROS are efficiently scavenged by ascorbate and glutathione, which decline after wounding but are still present at high concentrations. In the next chapters we will also present evidence suggesting that superoxide reacts with NO resulting in peroxynitrite formation and protein nitration.

Collectively, the presented data argue for wound-induced systemic redox signaling in the EFP. Future, work will reveal more details on the fate and functions of ROS during stress responses of the EFP.

### NO-mediated protein S-nitrosylation is decreased during the SWR

ROS, NO, and its oxo-derivatives cooperate in stress-induced redox signaling (Groß et al., 2013). Dependent on the redox status NO derivatives exhibit very different chemical properties. NO reacts with superoxide at high rate constant in the course of radical-radical scavenging resulting in the formation of ONOO^−^, which can oxidize and nitrate proteins (Pryor et al., 2006; Gaupels et al., 2011). Tyrosine (Tyr) and tryptophane residues are particularly amenable to nitration through attachment of a NO_2_ group. By contrast, NO itself and the derivative N_2_O_3_ rather modify peptides by cysteine (Cys) S-nitrosylation (NO group; Astier et al., 2012; Yu et al., 2014).

**Figure 3:**
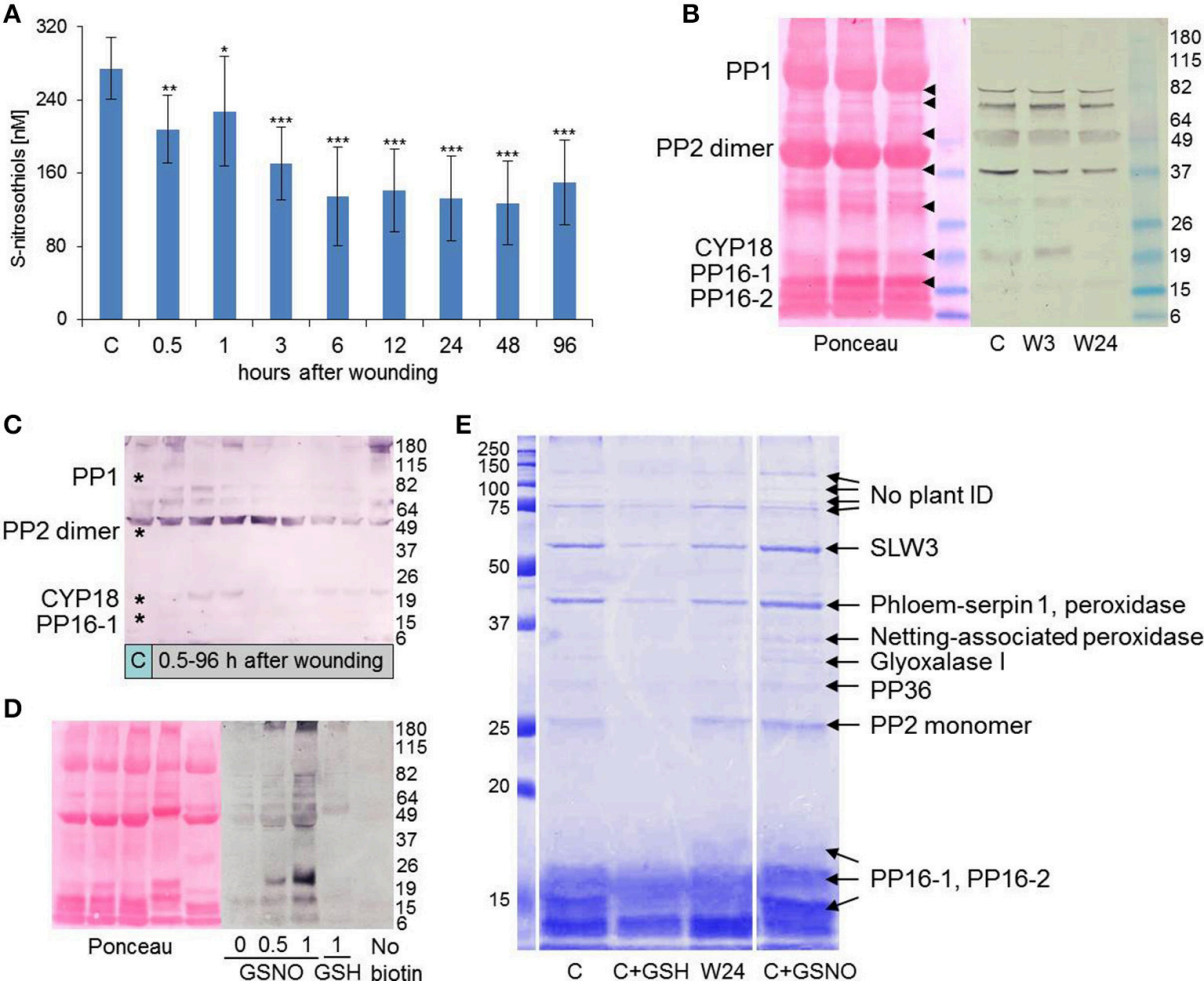
**Cysteine S-nitrosylation is reduced in the EFP after leaf wounding. (A)** Total S-nitrosothiols were determined using a Sievers Nitric Oxide Analyzer. Columns represent means (±SD, *n* = 7–11). Asterisks indicate significant differences from control (C) samples (Student's *t*-test, ^*^p < 0.05, ^**^p < 0.01, ^***^p < 0.005). **(B–D)** Anti-biotin western blot (WB) analyses for detection of S-nitrosylated EFP proteins after biotinylation of S-nitrosylated proteins by the biotin switch assay. Successful protein transfer was confirmed by Ponceau Red staining of the nitrocellulose membrane. Molecular weight (kD) of protein standards are indicated on the right. **(B)** Comparison of protein S-nitrosylation between phloem sap from untreated control pumpkin plants (C) and wounded plants (W3, W24, 3, and 24 h after wounding). Major phloem proteins are labeled (cf. Walz et al., 2004). Note that the non-reducing buffers used for the WB facilitated the formation of PP2 dimers (cf. Read and Northcote, 1983). Arrow heads indicate positions of WB signals on the Ponceau Red stained membrane. **(C)** Time course of protein S-nitrosylation after wounding. Asterisks mark the positions of major phloem proteins. **(D)** Effect of 0.5 and 1 mM GSNO and 1 mM GSH on S-nitrosylation. One non-biotinylated control is shown. **(E)** Isolation, separation and identification of S-nitrosylated EFP proteins. Proteins identified in cut bands are indicated. Phloem sap from control plants (C) was treated or not with 8 mM GSH or 8 mM GSNO for maximal protein yield. Note that S-nitrosylation is decreased in phloem sap from a wounded plant (W24). Protein standards are labeled on the left (kD).

The tripeptide glutathione has a Cys which is very sensitive to oxidation and is responsible for disulfide bond formation between two GSH molecules resulting in GSSG formation upon oxidation. This Cys is efficiently S-nitrosylated and S-nitrosoglutathione (GSNO) is a well-known reservoir of NO in plants and animals (Yu et al., 2014). NO homeostasis is under control of the enzyme GSNO reductase (GSNOR). In EFP samples S-nitrosothiol (SNO) levels were significantly down-regulated already at 30 min reaching about 50% of control levels at 6–48 h after leaf wounding (Figure 3A). The employed NO analyzer (NOA) cannot discriminate between protein- and low-molecular weight SNOs including GSNO. However, given the rather high GSH concentrations it can be assumed that GSNO accounts for a large fraction of total SNOs in the EFP.

For investigating protein S-nitrosylation we used the biotin switch method, which is based on replacing the SNO group by biotin. With anti-biotin antibodies it was then possible to detect candidate S-nitrosylated phloem proteins. In accordance with the NOA results, the biotin switch approach revealed a decline in S-nitrosylation of phloem proteins after wounding. Seven bands of approx. 16, 18, 29, 37, 50, 75, and 80 kD were visible in EFP samples from untreated pumpkin plants but gradually weakened at 3 and 24 h after leaf injury (Figure 3B). The 16- and 18 kD bands represent 16 kD Phloem Protein 1 (PP16-1) and Cyclophilin 18 (CYP18). Both proteins are wound-inducible in the EFP (Gaupels et al., 2012). The candidate S-nitrosylated ~50 kD protein is most likely an isoform of the Phloem Protein 2 (PP2) dimer. The dimer is stable in the non-reducing buffers used for the biotin switch technique, whereas mainly monomers of the PP2 isoforms are visible after reducing SDS-PAGE (Read and Northcote, 1983; Walz et al., 2004). A more detailed time course experiment revealed that S-nitrosylation was unchanged or even slightly increased at 0.5 and 1 h but clearly diminished between 6 and 48 h after leaf wounding (Figure 3C). Thus, at the later time points protein S-nitrosylation decreased with similar kinetics like the total SNO levels.

The specificity of the approach was validated by treating phloem samples with 1 mM GSH or 0.5 and 1 mM GSNO. GSH is a scavenger whereas GSNO is a donor of S-nitrosylation. Accordingly, basal S-nitrosylation in the EFP samples was strongly enhanced by GSNO but reduced by GSH pre-treatment (Figure 3D). The shift in band pattern upon GSH treatment probably reflects S-glutathionylation of PP16-1, CYP18, and PP2. Only very weak background signals were visible in the non-biotinylated control sample. It is noteworthy that the same set of proteins was S-nitrosylated *in vivo* as well as *in vitro* after GSNO treatment implying that these EFP proteins are major targets of NO binding.

Isolation of biotinylated proteins by streptavidin-coated magnetic beads facilitated the identification of candidate S-nitrosylated phloem proteins by mass spectrometry (MS) (Figure 3E, Supplemental Table 1). Also in this experiment S-nitrosylation decreased in EFP exudates from wounded pumpkin plants and in GSH-treated samples but increased after addition of GSNO. We used 8 mM GSNO in order to maximize protein yields before further analysis by MS. Except for PP16-2 all 9 identified proteins had at least one Cys residue as a prerequisite for S-nitrosylation. PP16-2 shares 91% sequence homology with PP16-1 but lacks the two Cyss near the N-terminus. Accordingly, PP16-2 was never detected in anti-biotin western blots (WBs). For the streptavidine-based pulldown of SNO-proteins we used high-salt but otherwise non-denaturating conditions. Therefore, it is plausible that PP16-2 was co-isolated with a biotinylated protein—most likely PP16-1. Alternatively, non-biotinylated PP16-2 efficiently binds to streptavidine in an unknown fashion. PP16 proteins are phloem-mobile RNA carriers (Xoconostle-Cázares et al., 1999). Therefore, S-nitrosylation of PP16-1 could affect RNA signaling in the phloem.

Both in the anti-biotin WB as well as in the streptavidine-pulldown a 26.5 kD rather than the major 24.5 kD isoform of PP2 was primarily modified by NO. Although, PP2 isoforms were described earlier (Read and Northcote, 1983; Walz et al., 2004) it is still not clear if they differ in sequence or in post-translational modifications. PP2 has 6 Cys and our data imply that at least one Cys is more amenable to S-nitrosylation in the 26.5 kD isoform compared to the 24.5 kD isoform. Reported functions of PP2 are related to the polygalacturon-binding lectin domain, RNA transport and redox-controlled formation of phloem filaments together with PP1 (Read and Northcote, 1983; Dinant et al., 2003). If these functions are modulated by S-nitrosylation remains to be investigated in a future study. Peroxidases are further redox sensitive proteins in the EFP. Both type III phloem peroxidases identified by the biotin switch technique contain several Cys residues within or near the catalytic center, which could play a role in regulation of enzyme activity by redox modifications including S-nitrosylation. To date, nothing is known about a possible NO-related regulation of the EFP proteins glyoxalase I, SLW3 (β-glucosidase), serpin-1 (proteinase inhibitor), and PP36 (cytochrome b5 reductase).

CYP18 was not identified in the streptavidin-pulldown probably because it is strongly expressed only at 0.5–6 h after wounding. No clear 18 kD band was visible at the 24 h time point and only a single peptide of CYP18 was found by MS (Figure 3E, Supplemental Table 1). In contrast, S-nitrosylated CYP18 was reproducibly detected by anti-biotin-WB at early time points after wounding and after GSNO treatment (Figures 3B–D). CYP18 has conserved Cys residues at sequence position 40 and 168. Disulfide bond formation between these Cys in the citrus (*Citrus sinensis*) cyclophilin CsCYP disrupted peptidyl-prolyl *cis-trans* isomerase activity and binding to the specific interaction partners thioredoxin and RNA polymerase II, thereby modulating gene transcription (Campos et al., 2013). S-nitrosylation of plant cyclophilins has been reported for CYP20-3 of Arabidopsis (Lindermayr et al., 2005) further supporting the assumption that S-nitrosylation of Cys-40 and -168 could impact on CYP18 functions during the SWR.

Mechanical injury triggered a rise in total SNOs in leaves of pea (*Pisum sativum*) and hypocotyls of sunflower (*Helianthus annuus*; Corpas et al., 2008; Chaki et al., 2011). GSNO levels were concomitantly increased in vascular tissues as visualized by immuno-localization with anti-GSNO antibodies. Contrary to these findings high basal SNO levels and protein S-nitrosylation were systemically decreased in the EFP after leaf wounding. High SNO levels could be explained by the hypoxic conditions prevailing in the phloem, which is deeply embedded in other tissues (van Dongen et al., 2003). Oxygen deficiency is a well-known stimulus for NO production (Perazzolli et al., 2004). The damage-induced reduction in SNO abundance could be caused either by down-regulated production or increased degradation of NO/SNO within the EFP. Preliminary analyses of rape seed (*Brassica napus*) phloem exudates confirmed the wound-induced decrease in protein S-nitrosylation suggesting that this stress response is not specific for the pumpkin EFP.

### Protein tyrosine nitration is enhanced during the SWR

**Figure 4:**
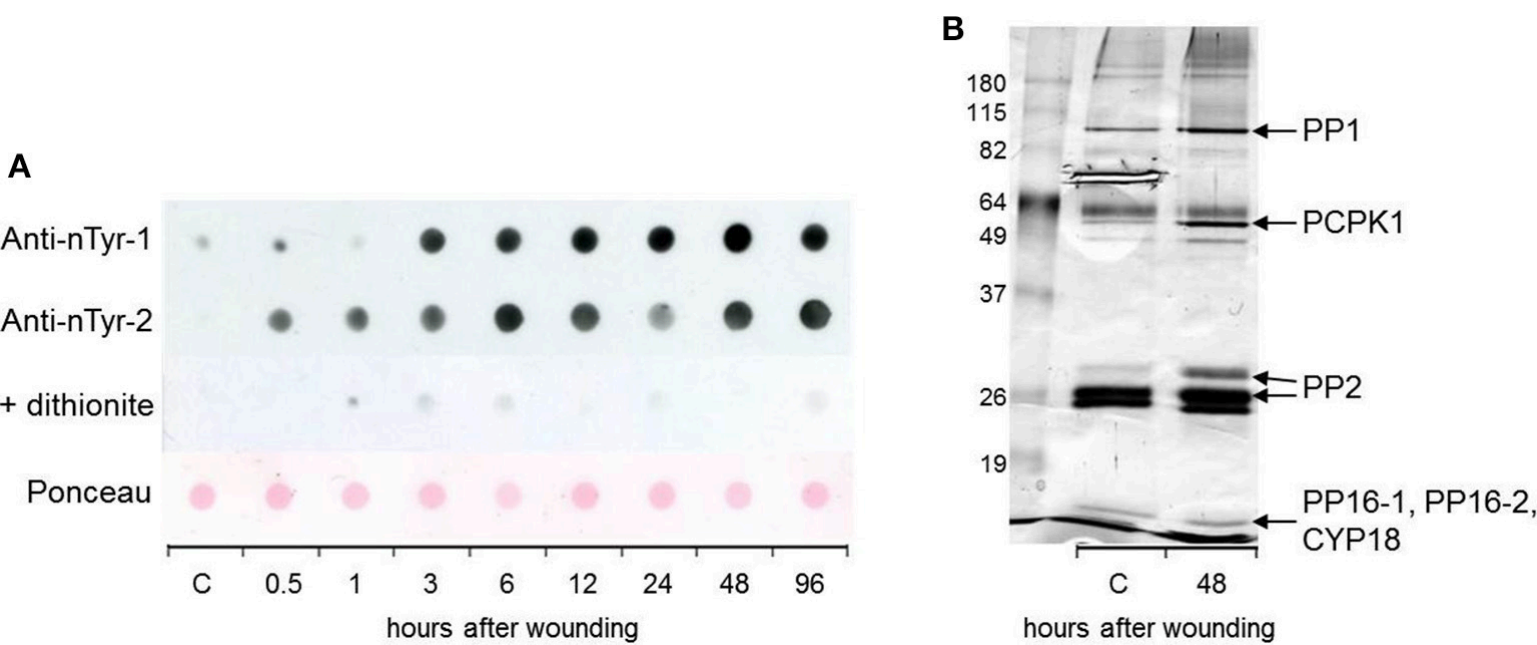
**Tyrosine nitration of EFP proteins is enhanced in response to leaf damage. (A)** Nitration was detected by anti-nitrotyrosine antibodies after dot-blot transfer of total EFP proteins to a nitrocellulose membrane. Two representative experiments are shown. A duplicate membrane of experiment 2 was incubated in 100 mM dithionite for de-nitration. Ponceau Red staining of the same membrane confirmed that phloem proteins were still present after dithionite treatment. **(B)** Immuno-precipitation and identification of tyrosine-nitrated EFP proteins. Proteins were silver-stained. C, control.

According to a current model Tyr nitration is a highly efficient radical-radical reaction between NO_2_ and ROS-generated protein Tyr radicals (Pryor et al., 2006). Thus, nitroTyr formation is indicative for the simultaneous presence of NO and ROS in stressed tissues. Protein Tyr nitration can be immuno-detected by anti-nitroTyr antibodies. However, nitration is a rare event and was only occasionally detected in EFP exudates by WB analysis after SDS-PAGE. Therefore, we analyzed cumulative nitration of phloem proteins after dot blot onto a nitrocellulose membrane (Figure 4A). Nitration of EFP proteins gradually increased peaking at 48 h after leaf wounding. In some experiments nitrated proteins accumulated already at the 0.5 and 1 h time points. Pre-incubation of the WB membrane with the de-nitrating compound dithionite before immuno-detection by an anti-nitroTyr-antibody resulted in almost completely abolished WB signals although phloem proteins were still present on the membrane as confirmed by Ponceau Red staining. This demonstrates that the employed monoclonal antibody was specific for tyrosine nitration. Using the same approach we observed nitration of EFP proteins after watering of plants with H_2_O_2_ (Gaupels et al., 2008) as well as after UV-B-, ozone-, and salt stress (unpublished results). Therefore, it can be speculated that ONOO^−^/NO_2_-mediated nitration is involved in the general stress response of the EFP.

For enrichment, isolation and subsequent identification of nitrated phloem proteins we employed an anti-nitroTyr antibody coupled to agarose beads. This way, we identified the candidate nitrated phloem proteins PP16-1/-2, CYP18 (detected in the same cut band) PP2, Phloem Calmodulin-like-domain Protein Kinase 1 (PCPK1), and PP1 (Figure 4B, Supplemental Table 2). Tyr nitration of the latter three proteins was enhanced at 48 h after leaf damage whereas the band intensity for PP16-1/-2 and CYP18 was not strongly altered. CDPK1 is a particularly interesting protein because it has been shown that various kinases can be regulated by Tyr nitration. For instance, human Protein Kinase G-1α was inhibited by nitration of Tyr247 whereas the activity of Protein Kinase Bα was enhanced after nitration of Tyr350 (Rafikov et al., 2013; Aggarwal et al., 2014). Tyr nitration was observed in various plants exposed to biotic as well as abiotic stresses including mechanical injury (Corpas et al., 2008; Chaki et al., 2011). However, physiological effects of protein nitration in wounded plants were not yet investigated.

### Leaf wounding triggers cGMP signaling in the EFP

**Figure 5:**
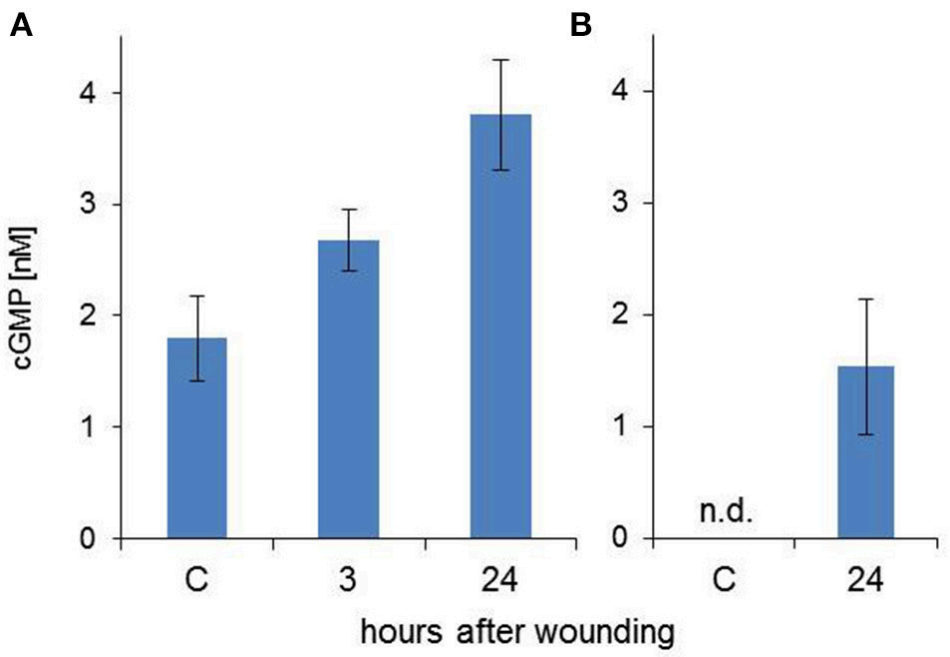
**Wounding induces cGMP signaling in the EFP**. cGMP concentrations were measured using a commercial ELISA kit **(A)** or by a HPLC-MS/MS system **(B)**. Columns represent means (±SD, *n* = 7 for **A**, *n* = 3 for **B**).

In response to stress cGMP is produced by soluble or membrane/receptor guanylate cyclases (GCs) (Gaupels et al., 2011). Two independent methods were employed for determination of cGMP levels in EFP exudates—a commercial enzyme linked immunosorbent assay (ELISA) kit based on specific antibodies against cGMP and a high performance liquid chromatography (HPLC)-MS/MS system optimized for measurements of cyclic nucleotides (Bähre and Kaever, 2014). ELISA is very sensitive and allowed for the detection of 1.8 nM basal cGMP levels, which increased to 2.7 nM at 3 h and 3.8 nM at 24 h after wounding (Figure 5A). The induction of cGMP signaling was confirmed by HPLC-MS/MS. cGMP control samples were below the detection limit of this technique but reached 1.5 nM at the 24 h time point (Figure 5B). Hence, both measurements showed that cGMP was present in the EFP at low nanomolar concentrations and cGMP synthesis was systemically up-regulated after leaf damage.

Injured leaf tissues undergo programmed cell death for protecting the plant from microbial ingress. Experiments with pelargonium and sweet potato (*Ipomoea batatas*) revealed a dual role of NO in onset and confinement of wound-induced cell death. Suicide is initiated by combined enzymatic ROS production and NO-mediated inhibition of antioxidant enzymes (Arasimowicz et al., 2009). However, tissues near the damaged leaf area were protected from cell death by enhanced expression of APX and superoxide dismutases, which was dependent on NO and down-stream signaling by cGMP as uncovered by use of GC inhibitors (Lin et al., 2011). Independent of NO cGMP induces expression of the microRNA miR828 after leaf wounding (Lin et al., 2012). cGMP production by soluble GCs in animal cells is activated by binding of NO to the protein haem domain through metal nitrosylation. In plants, the Arabidopsis NO-inducible GC 1 (NOGC1) is involved in regulating the stomatal aperture. The available literature shows that cGMP is not always down- but can also be up-stream of NO and ROS in plant stress responses (Gaupels et al., 2011). For instance, the oxidative burst and subsequent induction of genes coding for antioxidant enzymes was suppressed by an inhibitor of cGMP synthesis in cadmium-stressed pea plants (Romero-Puertas et al., 2007). The exact mode of interaction of cGMP with NO and redox signals in the EFP will be deciphered in a future study.

## Concluding remarks

**Figure 6:**
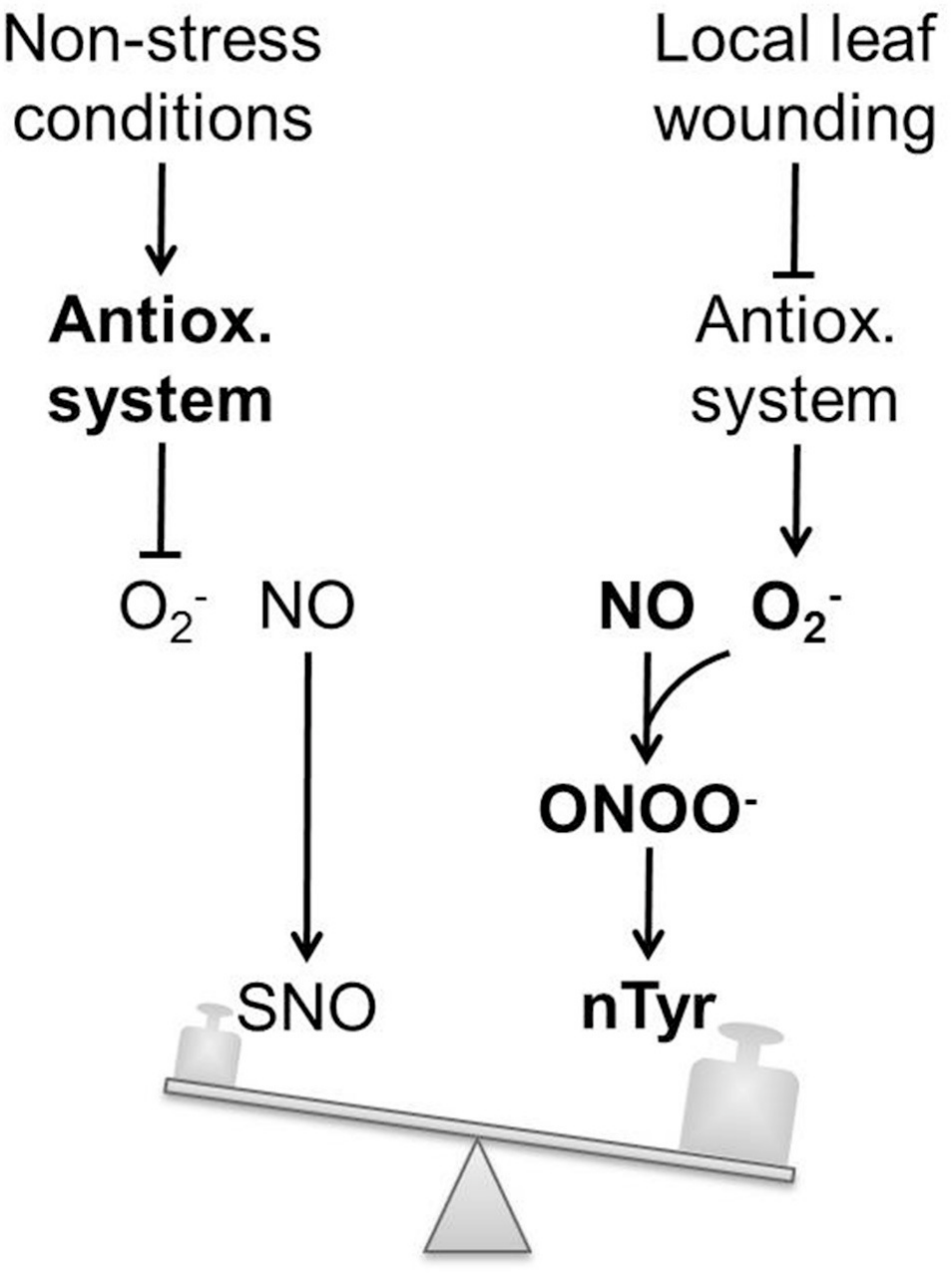
**Balance model of wound-induced NO- and redox-signaling in the EFP**. Under non-stress conditions low levels of O_2_- levels are maintained by the antioxidant system. Basal NO production in the EFP causes protein S-nitrosylation. Local leaf wounding triggers a systemic inhibition of the antioxidant system. Subsequent simultaneous accumulation of O${}_{2}^{-}$ and NO facilitates ONOO^−^ formation and tyrosine nitration. Hence tyrosine nitration is an indirect evidence for both O${}_{2}^{-}$ as well as NO synthesis within the pumpkin EFP. O${}_{2}^{-}$ has a very high affinity for NO. Therefore, tyrosine nitration is favored over S-nitrosylation after wound-induced O${}_{2}^{-}$ accumulation. SNO, S-nitrosothiols; nTyr, nitrotyrosine.

Leaf damage triggered a systemic wound response in the pumpkin EFP. Within 30–60 min the core antioxidant system of the EFP was rapidly down-regulated. We hypothesize that this would likely promote the accumulation of ROS such as O${}_{2}^{-}$ and H_2_O_2_ (Figure 6). NO-dependent protein modifications appeared to be altered with somewhat slower kinetics. The total SNO content and protein S-nitrosylation decreased whereas protein Tyr nitration increased after leaf injury. This finding cannot be explained by a general regulation of NO production or degradation. More likely, severe stress induced a shift in the composition of reactive nitrogen species away from NO to its nitrating oxo-derivatives ONOO^−^ and NO_2_. In such a scenario leaf wounding causes a drop in the EFP's antioxidant capacity and in subsequent accumulation of ROS. Particularly, O${}_{2}^{-}$ would efficiently scavenge NO to give ONOO^−^. This reaction has a higher rate constant than the binding of NO to GSH or protein thiols. Therefore, nitration would be favored over S-nitrosylation under oxidative stress conditions (Figure 6). Increased protein nitration is also an indirect evidence for the simultaneous accumulation of ROS (facilitated by the inhibition of the antioxidant system) and NO. Another outcome of our work is that PP16-1, CYP18, and the 26.5 kD isoform of PP2 are redox-sensors in the EFP, which can be carbonylated, S- glutathionylated, S-nitrosylated, or tyrosine nitrated. Hence, dependent on the levels and composition of redox signals in the EFP proteins are modified by different mechanisms with specific implications for protein conformation and function. Future, work will focus on deciphering the interactions between the antioxidant system, NO and cGMP as well as the effect of redox modifications on phloem proteins.

## Author contributions

FG, AF, MZ, and FC analyzed the antioxidant system. FG, AB, JK, and HS identified NO-modified proteins. VK measured cGMP. FG, KK, and JD planned the experiments. FG wrote the manuscript.

### Conflict of interest statement

The authors declare that the research was conducted in the absence of any commercial or financial relationships that could be construed as a potential conflict of interest.

## Supplementary material

Supplemental Figure 1 **Lipid oxidation as an indicator for oxidative stress does not increase after leaf wounding**. Lipid oxidation in EFP exudates was determined by the TBARS assay. Columns represent means (±SD, *n* = 9).

Supplemental Table 1 **Identification of candidate S-nitrosylated proteins in EFP latex**.

Supplemental Table 2 **Identification of candidate tyrosine nitrated proteins in EFP latex**.
