# Corrigendum: Systemic Induction of NO-, Redox-, and cGMP Signaling in the Pumpkin Extrafascicular Phloem upon Local Leaf Wounding

**DOI:** 10.3389/fpls.2016.00281

**Published:** 2016-03-07

**Authors:** Frank Gaupels, Alexandra C. U. Furch, Matthias R. Zimmermann, Faxing Chen, Volkhard Kaever, Anja Buhtz, Julia Kehr, Hakan Sarioglu, Karl-Heinz Kogel, Jörg Durner

**Affiliations:** ^1^Institute of Biochemical Plant Pathology, Helmholtz Zentrum München, German Research Center for Environmental HealthNeuherberg, Germany; ^2^Institute of General Botany and Plant Physiology, Friedrich-Schiller-UniversityJena, Germany; ^3^College of Horticulture, Fujian Agriculture and Forestry UniversityFuzhou, China; ^4^Research Core Unit Metabolomics, Hannover Medical SchoolHannover, Germany; ^5^Department Lothar Willmitzer, Max Planck Institute of Molecular Plant PhysiologyPotsdam, Germany; ^6^Biocenter Klein Flottbek, University HamburgHamburg, Germany; ^7^Department of Protein Science, Helmholtz Zentrum München, German Research Center for Environmental HealthNeuherberg, Germany; ^8^Research Center for BioSystems, Land Use and Nutrition, Institute of Phytopathology, Justus Liebig University GiessenGiessen, Germany

**Keywords:** phloem, systemic, wound response, signaling, NO, redox, antioxidant system, cGMP

In our original research article, there was a mistake in the fourth sentence of the abstract as published. Rather than “ascorbate reductase” (which does not exist) we investigated the enzyme activity of “ascorbate peroxidase.” The authors apologize for the mistake. This error does not change the scientific conclusions of the article in any way.

## Author contributions

All authors listed, have made substantial, direct and intellectual contribution to the work, and approved it for publication.

### Conflict of interest statement

The authors declare that the research was conducted in the absence of any commercial or financial relationships that could be construed as a potential conflict of interest.

