# Supplementary figures and images for: Systemic Induction of NO-, Redox-, and cGMP Signaling in the Pumpkin Extrafascicular Phloem upon Local Leaf Wounding
Source: Front Plant Sci. 2016 Feb 12;7:154. doi: 10.3389/fpls.2016.00154 (PMC4751408; doi:10.3389/fpls.2016.00154)

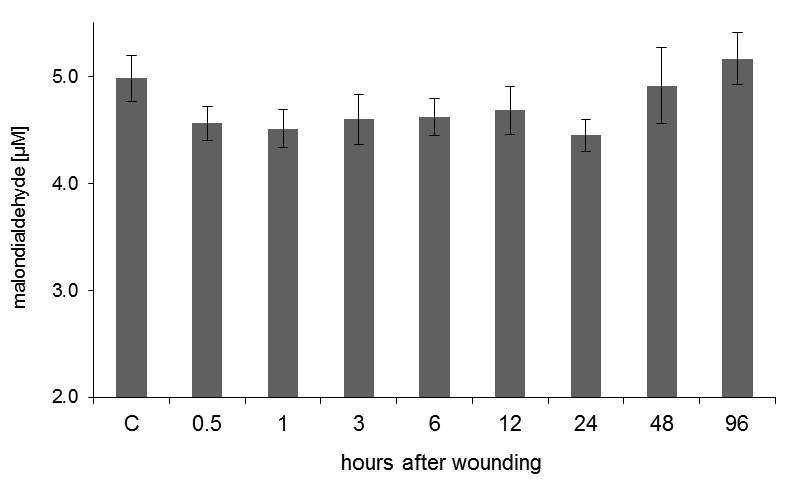

Supplement: Supplemental Figure 1 — Lipid oxidation as an indicator for oxidative stress does not increase after leaf wounding. Lipid oxidation in EFP exudates was determined by the TBARS assay. Columns represent means (±SD, n = 9). [file Image1.JPEG]
